# Performance evaluation of industry-education integration in higher education from the perspective of coupling coordination-an empirical study based on Chongqing

**DOI:** 10.1371/journal.pone.0308572

**Published:** 2024-09-18

**Authors:** Xianwen Gong

**Affiliations:** School of Economics and Business Administration, Chongqing University of Education, Chongqing, China; Air Force Engineering University, CHINA

## Abstract

The coupling coordination between higher education and regional industries is an effective way to enhance the performance of industry-education integration and an important driving force for promoting sustainable economic and social development. This article aims to evaluate the performance level of industry-education integration in higher education from the perspective of coupling coordination between industry and education, and to analyze its sustainable development trends. To achieve this, a framework for the industry-education complex system is constructed based on the theory of coupling coordination, and a performance evaluation model for industry-education integration is established. The "structure-conduct-performance" (S-C-P) analysis paradigm of industrial organization theory is used to design a performance evaluation indicator system, and the entropy weight method is applied to assign weights to the indicator system. Empirical research is conducted on Chongqing based on statistical data from 2000 to 2022. The results show that: (1) During the sample period, the construction effectiveness of industry-education integration in higher education in Chongqing is relatively significant, and the coordination level between regional industries and higher education continues to rise. (2) The orderly development level of the two subsystems of regional industries and higher education alternately rises, jointly promoting the continuous improvement of the performance level of industry-education integration, which is the main driving factor for performance improvement. (3) The stagnation of the coupling strength between regional industries and higher education is a key obstacle to further improving the performance of industry-education integration. Industry-education integration is a systematic project that should be promoted collaboratively from the perspectives of the regional industrial subsystem, higher education subsystem, industry-education complex system, and external environment to continuously enhance the performance level.

## 1. Introduction

The industry-education integration and the cooperation between schools and enterprises are effective ways to cultivate high-quality applied talents and promote technological innovation [1]. They are also important means to deepen higher education reform and promote sustainable development of education in the new era [2]. They play a significant role in promoting close cooperation among schools, enterprises, and the government, as well as sustainable development of regional economy and society [3]. Taking Foster’s "industry-university cooperation theory" as a representative, it regards industry-university cooperation as a kind of applied talent cultivation mode different from ordinary higher education, advocating that schools and enterprises should work together to solve the problem of "misalignment between student education and enterprise needs" [4], jointly run schools, jointly cultivate talents, and take the path of "industry-university cooperation" [5], which lays the theoretical foundation for the industry-education integration. The industry-education integration has characteristics such as combining engineering with learning, mutual participation, and serving society. Education should not be limited to traditional classroom teaching mode [6], encouraging close connection between educational activities and social production activities [7], cultivating talents needed for economic and social development [8, 9], and promoting sustainable industrial development [10]. The industry-education integration is the result of cooperation among schools, enterprises, and the government, which is affected by various factors from these entities [11]. It requires building a good partnership to achieve resource sharing and mutual complementarity [12]. Through the establishment of school-enterprise cooperation mode, various resources are optimized to form a talent cultivation mechanism for collaborative education [13].

In the practice of industry-education integration, represented by Dewey’s "learning by doing" theory, it emphasizes that the teaching process is a "process of doing", focuses on the combination of "doing" and "learning" in the talent cultivation process, and requires replacing the dominant position of traditional textbook-style textbooks with active, experiential and active assignments, connecting theory with practice. There are various cooperation models for open innovation between higher education and industry, such as industry-university-research cooperation, joint research and development, technology transfer, etc. [14, 15]. Several representative models with widespread influence have been formed: firstly, the enterprise-led model, which focuses on the dominant position of enterprises in industry-education integration. The most influential ones include Germany’s "dual system", Japan’s "enterprise visit" and South Korea’s "industry-education integration" system. Secondly, the school-led model, which highlights the role of schools in industry-education integration. Typical examples include France’s "apprenticeship training system" and Australia’s "new apprenticeship system". Thirdly, the equal emphasis on school and enterprise model, which is represented by the United States’ "contractual cooperation" and the United Kingdom’s "work-study alternating" model. It emphasizes the combination of school teaching and enterprise needs, actively cultivating students’ core technical skills.

In light of China’s actual situation, the reports of the 19th and 20th National Congresses pointed out that priority should be given to the development of education, deepen the industry-education integration, and build a high-quality education system. In order to implement the spirit of the industry-education integration, the central and local governments have introduced relevant policies, requiring deepening the industry-education integration, cultivating a large number of skilled talents, and integrating the industry-education integration as an important measure to promote coordinated economic and social development throughout the entire process of talent development. There is a complex coupling and coordination relationship between higher education, industry, and regional environment [16]. Taking the industry-education integration as the development direction, promoting the all-round integration of structural elements on the supply side of higher education and the demand side of industrial development, collaborating in school-enterprise cooperation to cultivate a large number of high-quality applied talents. Under the guidance of policies, the industry-education integration has attracted widespread attention from all walks of life. More and more enterprises have joined in the practice of collaborative education through the industry-education integration, actively exploring various forms of school-enterprise cooperation models.

Performance evaluation is the "baton" for the industry-education integration, which is related to the development direction of the industry-education integration. In December 2017, the General Office of the State Council issued "Several Opinions on Deepening the Integration of Industry and Education", which clearly stated that "actively support social third-party institutions to carry out performance evaluation of the industry-education integration, improve the statistical evaluation system. Strengthen the use of monitoring and evaluation results as an important basis for performance assessment, investment guidance, pilot implementation, and commendation and incentives". In January 2019, the State Council issued "the National Plan for the Reform and Implementation of Vocational Education", which pointed out that "improve the quality evaluation mechanism jointly participated by the government, industries, enterprises, vocational institutions, and actively support third-party institutions to carry out assessments". In October 2020, the CPC Central Committee and the State Council issued "the Overall Plan for Deepening Education Evaluation Reform in the New Era", which regarded the industry-education integration and cooperation between schools and enterprises as the key content of higher education performance evaluation, further highlighting the importance of performance evaluation of industry-education integration, and pointing out the direction for improving vocational education evaluation system.

The academic community home and abroad has actively explored the performance and evaluation of industry-education integration, with different opinions. Some scholars believe that the performance of industry-education integration is reflected in many aspects. University-industry cooperation promotes resource complementarity, which can bring scientific research operating funds and advanced experimental equipment to universities, and promote the improvement of scientific research performance. For example, Abramo et al. used bibliometric methods to investigate the scientific research performance of university-industry collaborations between Italian universities and domestic industries. Through empirical testing, they found that researchers involved in university-industry collaborations achieved better scientific research performance [17]. Meyer Krahmer and Schmoch’s research found that cooperation between enterprises and universities has significantly increased, but the interaction patterns in different technological fields are not consistent. For traditional university teaching and research activities, external resources such as funding support, creative sources, and experimental equipment can be obtained through school enterprise cooperation, which can help improve performance [18]. By integrating modern science and technology such as deep learning into products, it can effectively promote the improvement of product technology level [19]. Some scholars also believe that in the industry-education integration, the goals of schools and enterprises are not the same. Universities pursue collaborative education, while enterprises pursue economic benefits. There are contradictions and interests in school-enterprise cooperation, which do not significantly affect performance. In some cases, it may even have a negative impact on performance. For example, D’Este et al. conducted a study using relevant data from British universities and found that there was no significant positive or negative relationship between school enterprise cooperation and university research performance [20]. Rentocchini et al. used propensity score matching estimation methods to study the cooperation between schools and enterprises in five universities in Spain, and found that the cooperation between schools and enterprises was negatively correlated with the overall construction effectiveness [21].

Domestic research divides the performance of industry-education integration into three levels: macro, meso, and micro. The macro level focuses on the national level, focusing on issues such as the implementation effect of the industry-education integration policies, regional level differences, etc. For example, Liu et al. used the entropy method, coupling coordination degree model, and Tobit regression analysis model to study the spatial temporal evolution and influencing factors of production-education integration by constructing an evaluation index system for production-education integration in higher vocational education [22]. Luo et al. analyzed the effectiveness of the implementation of the industry-education integration policies in China’s vocational education, and proposed the construction of an effective monitoring system for the implementation effect of the industry-education integration policies [23]. Chen et al. evaluated the performance of industry-education integration from the perspective of resource integration, and found that the industry-education integration in higher vocational colleges has achieved some results, but the overall level of performance has yet to be improved, and the imbalance of performance has gradually become prominent, which requires the joint action of multiple paths such as policy guidance, operation optimization and market drive [24]. Xu et al. divided the integration process of industry and education into resource construction stage and achievement output stage. Based on the network DEA model to evaluate the effectiveness of the industry-education integration in China’s "Double High Plan" institutions, it was found that the overall operational efficiency of the industry-education integration was at a medium level, with a certain degree of differentiation among institutions. The comprehensive efficiency of institutions in the eastern, western, central, and northeastern regions decreased sequentially. There were obvious regional characteristics in the integration of industry and education in institutions [25].

At the mesoscopic level, the research focuses on the scientific research output and innovation achievements of the industry-education integration, as well as the cooperation between enterprises and universities. For example, Cai et al. studied the impact of industry-university cooperation on university innovation performance in the Chengdu-Chongqing dual-city economic circle. Through regression analysis, it was found that industry-university cooperation had a significant positive effect on university innovation performance [26]. Wang et al. used panel data from 88 Chinese universities from 2007 to 2014 to empirically study the impact of university-enterprise cooperation activities on scientific research performance, as well as the moderating role of university type. It was found that the impact of university-enterprise cooperation on university scientific research performance was inverted U-shaped, and university type had a significant negative moderating effect on the inverted U-shaped curve relationship between university-enterprise cooperation and university scientific research performance [27]. Li conducted research on the logic and path of the construction of industry-education integration communities from the perspective of new productivity, pointing out that industry-education integration communities fully highlight the needs of industry enterprises, and are a new organizational form that can enhance industry development performance and support high-quality development of the industry [28].

Micro level focuses on individual institutions such as the effect of school-enterprise cooperation in joint schooling and collaborative education. For example, Zhu systematically constructed a performance evaluation index system for vocational school enterprise cooperation projects, and conducted performance evaluation using the school enterprise cooperation projects between the school and the enterprise as an example [29]. Zhou et al. constructed a performance evaluation index system for the industry-education integration in higher vocational education from three dimensions: guarantee conditions, organization and implementation, and cooperation results. Taking Jiangsu Tourism Vocational College as an empirical case, they used the gray clustering evaluation method for performance evaluation [30]. Cheng et al. used the FAHP fuzzy analytic hierarchy process to establish a performance evaluation model and design a performance evaluation index system for school-enterprise cooperation. Supporting the major of finance and commerce in their institution, they evaluated the performance of school-enterprise cooperation [31]. Yang et al. explored the collaborative education mechanism between universities and enterprises for applied undergraduate education from the perspective of the integration of production and education, and proposed measures to deepen the integration of production and education and strengthen cooperation between universities and enterprises [32].

These studies have broadened the academic viewpoint on performance evaluation, facilitating the industry-education integration. They offer valuable insights for refining and enhancing the evaluation system of higher education. Nevertheless, the majority of the focus has been on case studies within higher education institutions, with inadequate emphasis on comprehensive systematic research. While considerable attention has been given to assessing the supply side of higher education, lacking of focus on the demands stemming from industrial development. Furthermore, the emphasis has predominantly been on evaluating the final outcomes, neglecting the significance of resource input levels.

The integration of industry and education is a systematic project, not equal to a simple mixture or quantitative addition between industry and education, but the integration development trend of the elements between the industrial subsystem and the educational subsystem, which is from disorder to order, from chaos to consistency. The key to achieve results lies in whether they can complement each other, achieve coordinated development and produce a synergistic effect. The performance evaluation of the industry-education integration should not only consider the supply side of education, but also take into account the demand side of industry; it should not only focus on the output results, but also take into account the input levels. In view of this, the paper establishes the analysis framework of the industry education composite system based on the synergy theory, builds an industry-education integration performance evaluation model which could take into account the factors on both sides of the supply and demand, uses the "structure-conduct-performance" (S-C-P) analysis paradigm to establish the performance evaluation index system, reflecting the internal logic of the industry-education integration, and taking into account the comparison of input level and output effect. Taking production-education integration of higher education in Chongqing as an example, this paper makes an empirical study on performance evaluation, and puts forward systematic improvement strategies from the perspective of collaborative development, which is a positive exploration.

## 2. Research methods and indicator system

### 2.1 Model setting

Synergistics theory posits that an open, nonlinear, and far-from-equilibrium system, when the external control variables reach a certain threshold, can evolve into a new ordered structure through a sudden change triggered by random fluctuations [33]. The complex interactions within a composite system may produce synergistic effects, leading to a virtuous cycle and promoting the development of the composite system towards orderliness, or they may have negative effects, causing the system to develop towards disorder [34]. When the synergistic effects are well-utilized, the degree of orderliness is high, and vice versa.

Based on the synergy theory, the industry education composite system is constructed. Under the framework of the industry education composite system, the industry-education integration refers to the evolution and development trend of the elements of the industry subsystem and the education subsystem infiltrating, crossing, coupling and complementing each other in the process of achieving the common goal, from disorder to order, from chaos to consistency. From this perspective, the key to achieving good performance of the industry-education integration lies in the complementary advantages of industry and education, collaborative development, and the synergistic effect of 1+1>2. This requires the education sector and the industrial sector to rely on their respective advantageous resources, based on mutual trust contracts, to serve economic and social development as the starting point, to take mutual benefit and win-win as the driving force, and to take school enterprise cooperation, project cooperation, technology transfer and joint development as the carrier, to teach in production and produce in teaching [35], and to cooperate closely to cultivate people, forming an optimized combination and highly integrated mode [36]. The integration of production and education is the organic combination of "productive learning" and "learning production", "productive teaching" and "teaching production" [37]. The performance of the industry-education integration shows the interactive coupling and collaborative development of industry and education in terms of scale and structure at the macro level, and the collaborative education of school enterprise cooperation at the micro level.

In a complex system, coupling refers to the phenomenon that two or more systems or forms of motion influence each other through various interactions, and this coupling effect can be reflected by the degree of coupling. Collaboration refers to the harmony and consistency between systems or their components during their development and evolution, and this degree of collaboration can be described by the degree of collaboration. At the phase transition point, the internal variables of the system include fast relaxation variable and slow relaxation variable, and the slow relaxation variable, that is, the order parameter of the system, plays a decisive role in the phase transition process of the system. The synergistic effect between the order parameters within the system dominates the trend of the system’s evolution from disorder to order, and determines the characteristics and laws of the system’s phase transition. The degree of coupling and coordination determines the type of order and structure that the system will adopt when it reaches the critical region [38].

Based on the complex system of industry and education, a performance evaluation model for the industry-education integration is constructed from three aspects: the efficacy coefficient of order parameters, the order degree of subsystems, and the coupling collaboration degree of the complex system. Assume that the order parameter in the evolution process of the industrial education composite system is *u*_*i*_(*i* = 1,2), that *u*_*ij*_(*j* = 1,2,3,…,*n*) is any component of the order parameter *u*_*i*_, and that the value is *X*_*ij*_. The upper and lower limits of the order parameter are *α*_*ij*_ and *β*_*ij*_, respectively, and the contribution that this order parameter promotes the orderly development of the composite system can be expressed as

$$U(u_{ij})=\left\{\begin{array}{c} \frac{X_{ij}-\beta_{ij}}{\alpha_{ij}-\beta_{ij}},u_{ij}\;\text{has}\;\text{positive}\;\text{effect}\\ \frac{\alpha_{ij}-X_{ij}}{\alpha_{ij}-\beta_{ij}},u_{ij}\;\text{has}\;\text{negative}\;\text{effect} \end{array}\right.$$ (1)

In formula (1), *U*(*u*_*ij*_) is the order degree of the order parameter *u*_*ij*_, and *U*(*u*_*ij*_) ∈ [0,1]. The larger the value is, the greater the contribution of the order parameter to the system. The total contribution of the order parameter to the system can be achieved by integration, and the formula is as follows:

$$U(u_{i})=\sum_{j=1}^{n}\;\lambda_{ij}U(u_{ij})$$ (2)

In formula (2), *U*(*u*_*i*_) is called the order degree of the subsystem, *U*(*u*_*i*_) ∈ [0, 1], *λ*_*ij*_ is the weight of the order parameter, and $\lambda_{ij}\geq 0,\sum_{j=1}^{n}\;\lambda_{ij}=1$. The greater the order degree of the subsystem is, the higher the orderly development level of the system, and vice versa.

Referring to the concept of capacitive coupling and the model of capacity coupling coefficient in physics, the interaction coupling model of multiple systems (or elements) is generalized. The formula *C*_*n*_ = {(*u*_1_ ⋅ *u*_2_ ⋅ … ⋅ *u*_*m*_)/[∏ (*u*_*i*_ + *u*_*j*_)]}^1/*n*^ could represent the coupling degree under multisystem interaction conditions. Therefore, the coupling function of the industrial education composite system can be expressed as

$$C=\frac{{\left[U(u_{1})\cdot U(u_{2})\right]}^{1/2}}{U(u_{1})+U(u_{2})}$$ (3)

In formula (3), *C* is the coupling degree of the industrial education composite system, *U*(*u*_1_) is the order degree of the industrial subsystem, and *U*(*u*_2_) is the order degree of the education subsystem.

Obviously, *C* ∈ [0,1], and the greater the value of the coupling degree is, the higher the coupling strength. The coupling degree can better reflect the coupling strength between industry and education but cannot reflect the coordination level. Therefore, it is necessary to establish the following coupling coordination function to analyze the coordination degree of interaction coupling between industry and education.


$$\left\{\begin{array}{l} D={\left(C\cdot T\right)}^{1/2}\\ T=a\cdot U(u_{1})+b\cdot U(u_{2}) \end{array}\right.$$ (4)

In formula (4), *D* is the coupling coordination degree, *T* is the coordination indicator of industrial education, reflecting the effect and contribution of the comprehensive evaluation index of the two subsystems to their coordination degree, *a* and *b* are undetermined coefficients. In the process of coupling coordination development, industrial and education are supplementary and indispensable to each other and of equal importance, the coefficient values of this study are a = 0.5 and b = 0.5. The higher the coupling and collaboration degree, the better the system’s coupling and collaboration level. It is best to ensure this, as it can guarantee that the coupling and collaboration degree model reflects both the coupling intensity and collaboration level of industry-education integration.

### 2.2 Indicator system

In the 1930s, the Harvard School proposed the famous "Structure-Conduct-Performance" (S-C-P) analytical framework, which deeply analyzed the relationship between structure, conduct, and performance in the context of industrial competition, becoming one of the most influential analytical paradigms in the theoretical system of industrial economics. According to the S-C-P analytical framework, the basic logic of industrial development should be that during the process of industrial competition and development, the structure of industry determines the conduct of industry, and the conduct of industry in turn determines the performance of industry [39, 40]. According to this framework, in order to achieve ideal performance, industry and education can achieve economies of scale through expansion, promote growth in industry and education, enhance competitiveness in industry and education, and indirectly improve or directly intervene to optimize irrational structures in industry and education through public policies. Therefore, decomposing the indicator system from three aspects: structure, conduct, and performance is expected to reflect the intrinsic logical relationship achieved in the integration of industry and education.

#### (1) Industry subsystem

In terms of structure, industrial structure is also called sectoral structure of the national economy, which refers to the composition of the various industrial sectors and within each sector. The proportion of primary, secondary, and tertiary industries reflects the proportion of different industries in the national economy, and it is a typical indicator to measure industrial structure. According to the theory of industrial structure evolution, the main problem of industrial structure is the rationalization of industrial structure, that is, the coordination between industries, strong ability to transform industrial structure, adapt to changes in market demand, and produce good economic and social benefits. The overall proportion of primary, secondary, and tertiary industries in GDP reflects the proportion structure of each industry in the entire national economy. Adjusting industrial structure is a fundamental condition for China to achieve stable and sustainable economic development, a main way to improve economic efficiency, and an important factor to promote the transformation of economic growth mode. Meanwhile, adjusting and optimizing industrial structure is of great significance for expanding employment, improving people’s living standards, reducing energy and material consumption, reducing environmental pollution, improving ecological environment, transforming trade growth mode, and improving international competitiveness [41].

In terms of conduct, industrial scale reflects the increase or expansion in quantity in the development of industry and is the basic manifestation of the level of industrial development. The basic goal pursued by the development of industrial scale is economies of scale, that is, the cost of products decreases and returns increase with the expansion of production and business scale. The benefits brought by economies of scale are reflected in the reduction of the cost of production and operation of enterprises. Conversely, an unreasonable scale is not easy to produce economies of scale, which is not conducive to forming a competitive advantage, resulting in a serious waste of resources. Industrial scale is a basic factor that the state needs to consider when formulating industrial policies. The industrial scale is reflected by the scale of output value and the organization size of industries, and the level of industrial development and growth is reflected by investment growth, output value growth, and profit growth.

Industry performance is reflected by both input and output. Input refers to the sum of human resources, material resources and financial resources invested in the development of various industrial sectors of the national economy is the basic condition and material basis for industrial development. According to the life cycle theory of industrial development, the basic issue that should be considered in industrial investment is to adopt different investment levels and allocation strategies at different stages of its development, rationally allocate relatively scarce resources, produce the most suitable goods and services with the least resource consumption, and obtain the best benefits. Industry output refers to the final results of production and business activities of various industrial sectors of the national economy in a certain period and its contribution to economic and social development, which reflects the development efficiency of the national economy, embodies the increase of industrial economic benefits and the improvement of labor productivity. The level of industrial investment is comprehensively reflected by human resource investment, fixed asset investment, and various energy inputs, and the level of industry output is reflected by profitability and per capita output, forming the following indicator system. as shown in Table 1.

**Table 1:** Industry subsystem indicators.

Frame	Dimension	Index	Statistical calculations
Structure	Industry structure	The proportion of primary industry (c1)	Value of primary industry/total value × 100%
		The proportion of the secondary industry (c2)	Value of the secondary industry/total value × 100%
		Proportion of the tertiary industry (c3)	Value of the tertiary industry/total value × 100%
Conduct	Industry l scale	Value scale (c4)	Gross domestic product (GDP)
		Organizational size (c5)	Number of industrial enterprises above designated size
	Industry development	Investment growth (c6)	(Current investment—previous investment) / previous investment × 100%
		Value added (c7)	(Current period value—last period value) / last period value × 100%
		Profit growth (c8)	(Total profit in the current period—total profit in the previous period) / total profit in the previous period × 100%
Performance	Industry input	Human resource input (c9)	Employment in industry
		Fixed assets investment (c10)	Fixed assets investment of the whole society
		Energy inputs (c11)	Energy consumption
	Industry output	Level of profitability (c12)	Total profit of enterprises above the designated size
		Output per capita (c13)	GDP per capita

#### (2) Higher education subsystem

In terms of structure, the structure of higher education is closely related to the structure of industry and employment [42]. Optimizing the structure of higher education is an important means to build a high-quality education system and promote the high-quality development of education. The adjustment of higher education structure plays a promoting role in the optimization of the structure of primary, secondary and tertiary industries [43]. The proportion of education funds to GDP and the student-teacher ratio of higher education institutions reflect the structure of education funds and the structure of teachers and students in higher education. The proportion of public education funds to GDP reflects the degree of financial support provided by a country’s government departments for the development of education, which is one of the basic guarantee conditions for educational development. It is also an internationally accepted structural indicator for measuring educational security conditions. The student-teacher ratio reflects the structure and proportion relationship between the number of students and teachers in higher education, which is an important indicator for measuring the conditions and benefits of generalized schooling, and also one of the important indicators for international and regional comparisons in education.

The conduct aspect includes two dimensions: education scale and education development. The education scale mainly reflects the number of education institutions at all levels and types, the number of students, the number of schools, and their growth, which indicates the degree of development of education. The scale of education is restricted by the population and its age structure, the level of economic development and the level of scientific and technological development. An appropriate scale of education can produce higher economic and social benefits of education, and a certain scale of higher education is compatible with a certain level of economic development [44]. Educational development refers to the deepening and progress in educational funding, teacher strength growth, and school size improvement. The basic trend of world higher education development is the popularization, lifelong learning, informatization, and internationalization of higher education [45].

In recent years, China’s educational development capability has been significantly improved, with a historic breakthrough in educational investment. The per capita funding system has gradually been established, and the school conditions of all levels of schools, especially rural schools, have been greatly improved. The quality of the teaching staff has been further improved, and the overall promotion of education informatization has been achieved. Important progress has been made in the reform of the education system, and the reform of the talent cultivation system, school management system, evaluation system, and security system has been comprehensively deepened. Breakthroughs have been made in some key areas and links, which is the driving force for the long-term healthy development of China’s education. This paper focuses on the industry-education integration in higher education institutions. Using the school size and student size of higher education institutions to reflect the scale of higher education is somewhat representative. The per capita educational funds and educational infrastructure reflect the basic guarantee for the development of higher education. The growth of teaching staff, educational funds, and school scale reflects the development situation of higher education. The National Medium- and Long-Term Education Reform and Development Plan (2010–2020) has completed its historical mission, and China’s higher education scale and structure have achieved various set goals [46].

In terms of performance, educational performance reflects the efficiency of educational development, using both input and output to reflect higher education performance [47, 48]. Educational input refers to the various resources invested by the entire society in education, including human, financial, and material resources, which are the basic guarantee for educational development. From the perspective of the entire society’s education, good educational output means more opportunities for education and higher levels. The most representative indicators to reflect educational quality are the scale of higher education students per 100,000 population and the gross enrollment rate of higher education. The scale of higher education students per 100,000 population is one of the important determinants of the number of high-level professionals in a country’s future population, and it is also an important indicator of the average level of education among the employed population. Therefore, the number of residents with university degrees determines to some extent the potential and possibilities for a country’s future social development. The gross enrollment rate of higher education reflects the overall level of higher education opportunities provided by a country, which is closely related to factors such as a country’s level of economic development and the degree of development of basic education. It is a widely cited indicator in China’s higher education academic community. Therefore, using human resource input, fixed asset input, and various energy inputs to reflect the level of higher education input, the number of higher education students per 100,000 population and the gross enrollment rate of higher education together reflect the development achievements of higher education in a period of time, forming an education subsystem indicator system as shown in Table 2.

**Table 2:** Higher education subsystem indicators.

Frame	Dimension	Index	Statistical calculations
Structure	Education structure	Percentage of education funds (j1)	Education funding/GDP × 100%
		College student teacher ratio (j2)	Number of students in ordinary colleges and universities/number of teachers in ordinary colleges and universities
Conduct	Education scale	School size (j3)	The number of ordinary higher education schools
		Student scale (j4)	Number of students in regular institutions of higher learning
	Education develop	Per capita education funding (j5)	Educational funding/number of students in regular higher education institutions
		Educational infrastructure (j6)	The number of public libraries in the area
		Increase in faculty strength (j7)	(Number of teachers in the current period—number of teachers in the previous period) / number of teachers in the previous period × 100%
		Educational funding increase (j8)	(Current education funds—previous education funds) / previous education funds × 100%
		Growth in school scale (j9)	(Number of students in the current period—number of students in the previous period) / number of students in the previous period × 100%
Performance	Education input	Capital investment (j10)	Expenditure on education in local fiscal budget
		Teaching staff input (j11)	Number of full-time teachers in regular higher education
	Education output	Level of higher education (j12)	The number of students in higher education institutions per 100,000 population
		Higher education opportunities (j13)	Gross enrollment rate of higher education

### 2.3 Entropy weight model

The entropy weight method is an objective weighting method that decides the index weight through the information amount provided by the entropy value of each index. The use of the entropy weight method to weight each index can avoid the interference of human factors in the weight of each evaluation index and make the evaluation result more in line with reality. Generally, entropy refers to disorder or uncertainty. Shannon entropy was introduced by Claude E. Shannon in his 1948 paper, “A Mathematical Theory of Communication”. In information theory, entropy is a measure of the disorder degree of a system. The higher the order degree of a system is, the smaller the entropy, and the greater the amount of information contained in the system [49, 50]. According to the theory, Shannon entropy is defined as *H* = −*s* Σ *p*_*i*_ ln *p*_*i*_.

For a particular assessment problem, there is a raw data matrix *X* formed of *m* years and *n* indexes; the element *x*_*ij*_ in the matrix represents the raw data value of the year *i*(*i* = 1,2,…,*m*) to the index *j*(*j* = 1,2,…,*n*).


$$X=\left[\begin{array}{cccc} x_{11} & x_{12} & \ldots & x_{1n}\\ x_{21} & x_{22} & \ldots & x_{2n}\\ \ldots & \ldots & \ldots & \ldots \\ x_{m1} & x_{m2} & \ldots & x_{mn} \end{array}\right]$$ (5)

The raw data in matrix *X* must be standardized to eliminate the effects of different dimensions and magnitude, and then, these data can be used in entropy calculations. Standardization operation should utilize an appropriate calculation method in accordance with the entropy calculation and the nature of the indexes. Commonly used standardization methods, such as z-score and equalization, could eliminate the effects of different dimensions and magnitudes [51]. Therefore, the z-score method is not suitable for use because the data normalized by this method are distributed between -1 and 1, and there is a logarithm in the entropy calculations. The equalization method could not guarantee that the value of all processed data is positive, and thus, it is also not suitable for use. Range standardization is a suitable method here [52, 53], and its formula is as follows:

$$\left\{\begin{array}{c} r_{ij}=\frac{x_{ij}-\text{min}(x_{ij})}{\text{max}(x_{ij})-\text{min}(x_{ij})},\;\text{for}\;\text{positive}\;\text{indexes}\\ r_{ij}=\frac{\text{max}(x_{ij})-x_{ij}}{\text{max}(x_{ij})-\text{min}(x_{ij})},\;\text{for}\;\text{reverse}\;\text{indexes} \end{array}\right.$$ (6)

For positive indexes (the larger the superior), the formula above is suitable. For the reverse index (the smaller the superior), the following formula is suitable. The processing result forms the standardization matrix *R* = (*r*_*ij*_)_*m*×*n*_, 0 ≤ *r*_*ij*_ ≤ 1.

For an index, the greater the difference in the evaluation index values, the larger the amount of information contained in this index, and the greater the effect of this index. According to the concept of entropy, the increase in information means a reduction in entropy, and entropy can be used to measure the size of this information. The feature proportion of year i under index j can be defined as follows:

$$p_{ij}=\frac{r_{ij}}{\sum_{i=1}^{m}\;r_{ij}}$$ (7)

The Shannon entropy of index j can be defined as follows:

$$E_{j}=-k\sum_{i=1}^{m}p_{ij}\;\text{ln}\;p_{ij}\;j=1,2,3\ldots n$$ (8)

In the formula, *k* = 1/ln *m*, when *p*_*ij*_ = 0 and *p*_*ij*_ ln *p*_*ij*_ = 0. The entropy weight of index j can be defined as follows:

$$\omega_{j}=\frac{1-E_{j}}{n-\sum_{j=1}^{n}\;E_{j}}$$ (9)

In the formula, 0 ≤ *ω*_*j*_ ≤ 1, $\sum_{j=1}^{n}\;\omega_{j}=1$, and the characteristics of the entropy weight are as follows:

When the index values of each evaluated object are entirely equal, the entropy value is the maximum, and the entropy weight is zero. This means that the index does not provide any useful information for decision-makers; this index can be considered canceled.

When the index values of each evaluated object are quite different, the entropy value is smaller, and the entropy weight is large. This means that the index provides decision-makers with useful information, and this index should be focused on.

The larger the entropy value of the index is, the smaller the value of entropy weight, and the less important the index.

Entropy weight has special significance; it is not the actual importance factor of the given indexes in the process of decision or assessment but rather the relative intensity factor of each index in a competitive sense under the condition that each evaluation index value has been determined after a given set of evaluated objects.

From the information point of view, entropy weight represents the extent of useful information provided by the index in question [54].

## 3. Empirical research and results analysis

### 3.1 Research process

Firstly, empirical research is conducted based on the statistical data of Chongqing from 2000 to 2022, which are sourced from the "Chongqing Statistical Yearbook" from 2001 to 2023. The original statistical data of the "higher education gross enrollment rate" from 2000 to 2006 are missing, and linear interpolation is used to fill the gap.

Secondly, the indicator system can be divided into three types: positive indicators, negative indicators, and moderate indicators. Among them, negative indicators and moderate indicators need to be processed positively. Negative indicators include the "student-faculty ratio in higher education" in the education subsystem, which is processed positively by using the reciprocal method. There are three moderate indicators, namely the proportion of primary, secondary, and tertiary industries in the industrial subsystem. These three indicators are not necessarily the larger the better. They have a moderate development trend and goal, all of which belong to moderate indicators. The industrial structure of developed countries has already become the perfect "three-two-one" structural model. Internationally, taking the United States as a reference, the proportion of primary industry output value is very small, generally remaining at around 1%. However, agricultural production has always been in a state of low input and high efficiency. The proportion of the secondary industry remains at around 20%, and the proportion of industry in the GDP of the United States is 19%. The proportion of the tertiary industry is the highest, fluctuating around 80%, and it dominates in the United States economy [55]. The industrial structure of provinces and cities such as Beijing, Shanghai, and Guangzhou in China also shows this trend. The proportion of the tertiary industry is very high, such as 81.0% and 69.9% in Beijing and Shanghai in 2018 respectively. It is a general trend for Chongqing’s industrial structure to shift from the original "one-two-three" model to a "three-two-one" model. By 2018, the proportions of the "one-two-three" industries were 6.80%, 40.90%, and 52.30% respectively, which are close to the national overall levels of 7.20%, 40.70%, and 52.00%. In the future, the proportion of the primary and secondary industries will further decline. Taking into account internal and external factors and development trends, it is considered to set the targets for the tertiary industry structure at 5%, 35%, and 60% for optimization.

Thirdly, Due to the different dimensions and data sizes of each order parameter index, it must be standardized. The commonly used standardization methods include Z-score standardization and averaging, but neither of them can ensure that the standardized data is greater than 0, which cannot meet the requirements of the entropy weight method. The range standardization is adopted here. For the indicators that have been processed in the forward direction (the larger the better type indicator), *r*_*ij*_ = [*x*_*ij*_ − min(*x*_*ij*_)]/[max(*x*_*ij*_) − min(*x*_*ij*_)] is adopted for standardization.

Fourthly, the entropy weight method is used to weight the index system, which can better retain the original information of the index data and improve the objectivity of the evaluation. If the smaller the information entropy of an index, the greater the degree of variation of its index value, the greater the amount of information it provides, and the greater its role in the comprehensive evaluation, the greater its weight should be. On the contrary, the larger the information entropy of an index, the smaller the degree of variation of its index value, the smaller the amount of information it provides, and the smaller the role it plays in the comprehensive evaluation, the smaller the weight should be. The results are shown in Table 3.

**Table 3:** Entropy weight of indicators.

Subsystems	Index	C1	C2	C3	C4	C5	C6	C7
Industry subsystems	*E*_*j*_	0.8650	0.9113	0.8891	0.8884	0.8551	0.9235	0.8678
	*ω*_*j*_	0.0950	0.0624	0.0780	0.0785	0.1020	0.0538	0.0931
	Index	C8	C9	C10	C11	C12	C13	—
	*E*_*j*_	0.8648	0.8741	0.9379	0.9463	0.8394	0.8648	—
	*ω*_*j*_	0.0952	0.0886	0.0437	0.0378	0.1130	0.0952	—
Subsystems	Index	J1	J2	J3	J4	J5	J6	J7
Education subsystems	*E*_*j*_	0.8663	0.9330	0.9461	0.9299	0.9298	0.9006	0.9361
	*ω*_*j*_	0.1233	0.0618	0.0497	0.0647	0.0648	0.0917	0.0590
	Index	J8	J9	J10	J11	J12	J13	—
	*E*_*j*_	0.8774	0.9010	0.9616	0.9049	0.8828	0.9464	—
	*ω*_*j*_	0.1131	0.0913	0.0355	0.0877	0.1081	0.0495	—

### 3.2 Result analysis

Performance evaluation is conducted from both subsystem and composite system levels. At the subsystem level, the effectiveness coefficient of order parameters and the orderliness of subsystems are mainly calculated, with a focus on analyzing the development trend of the orderliness of industry and education subsystems. At the composite system level, the coupling degree and coupling synergy degree are mainly calculated, with a focus on analyzing the evolution trend of interactive coupling and collaborative development of the industry-education composite system.

#### (1) Performance and development trend of the education and production subsystem

The upper and lower limits of each order parameter increase or decrease by 5% respectively on the basis of the actual maximum or minimum value, and the above model is used to calculate the order parameter efficacy coefficient and subsystem order. According to synergism, the efficacy coefficient represents the contribution of order parameters to the orderly development of subsystems. The greater its value, the greater its contribution, and the higher the degree of orderly development, and vice versa. From the calculation results, it can be found that from 2000 to 2022, the order degree of Chongqing’s industrial subsystem and education subsystem increased steadily, and the overall trend was upward, which promoted the orderly development of the two subsystems. as shown in Table 4.

**Table 4:** Orderly development level of industry and education subsystem.

Year	2000	2001	2002	2003	2004	2005	2006	2007
Industry subsystem	0.2151	0.1388	0.1472	0.1587	0.1386	0.1252	0.1445	0.2040
Education subsystem	0.2478	0.3075	0.2818	0.2674	0.2655	0.2527	0.3092	0.3169
Year	2008	2009	2010	2011	2012	2013	2014	2015
Industry subsystem	0.2644	0.2692	0.3482	0.3654	0.3427	0.3970	0.4478	0.4929
Education subsystem	0.3293	0.3336	0.3642	0.4201	0.5266	0.4259	0.4546	0.4743
Year	2016	2017	2018	2019	2020	2021	2022	—
Industry subsystem	0.5431	0.5828	0.6063	0.6616	0.6509	0.7476	0.6928	—
Educational subsystem	0.4752	0.5026	0.5264	0.5795	0.6054	0.6105	0.6190	—

**Fig 1:**
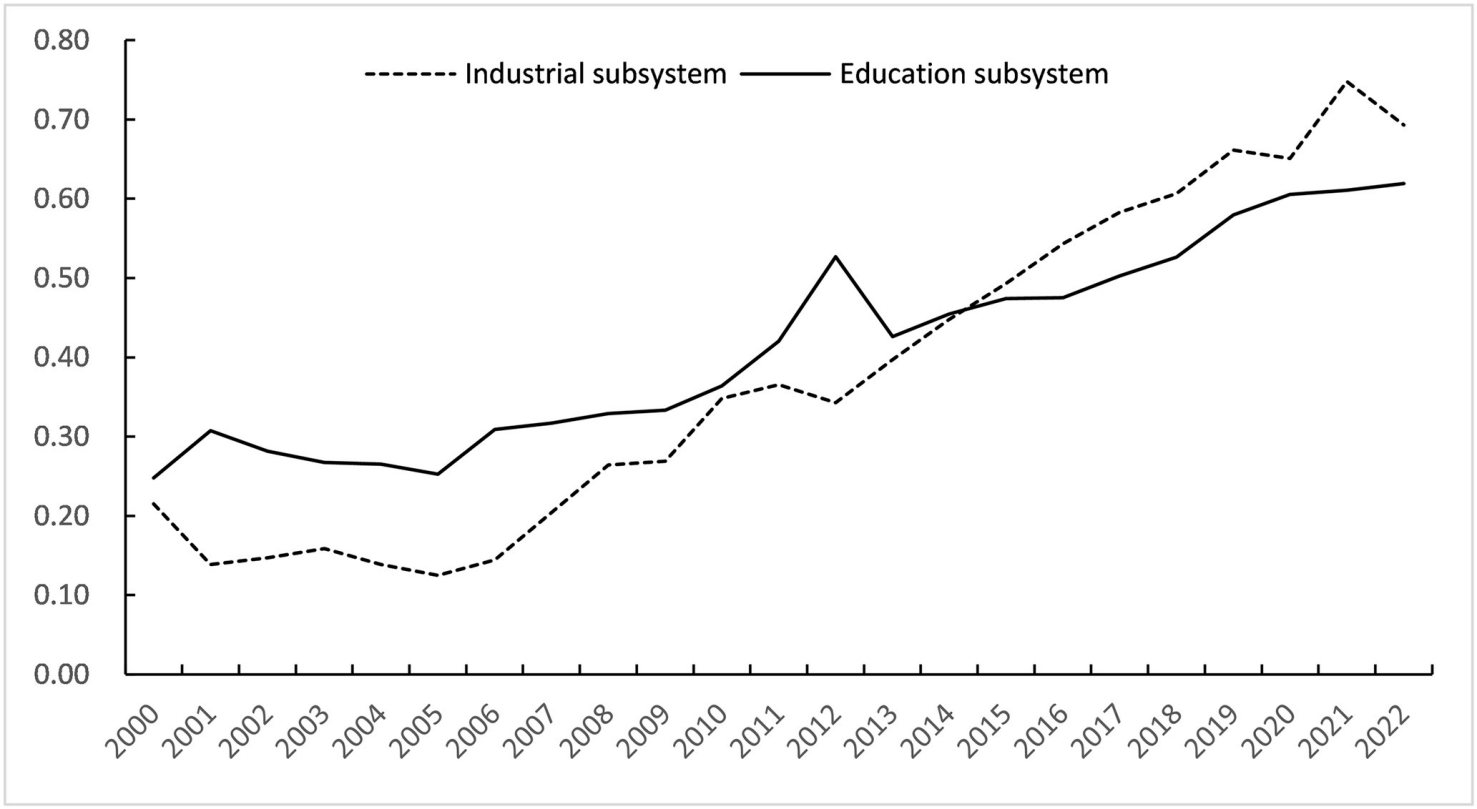
Orderly development trend of industry and education subsystem.

Overall, during the period from 2000 to 2022, the orderliness degree of the industrial subsystem educational subsystem in Chongqing’s industry-education composite system continued to rise alternately, which also jointly determined the continuous increase in the synergy of the composite system. From 2000 to 2022, the orderliness curves of the industrial and educational subsystems have been continuously rising alternately, indicating that the overall level of orderly development of industry and education in Chongqing is constantly improving. Among them, the orderliness of industrial development has increased from 0.2151 in 2000 to 0.6928 in 2022, with a significant increase in orderliness. The order degree of higher education has increased from 0.2478 in 2000 to 0.6190 in 2022, which is less than that of the industrial subsystem. It can also be observed that before 2014, the orderliness of industry was lower than that of education, indicating that during this period, industry development lagged behind education development, which restricted the improvement of the integration level between industry and education. After 2014, the orderliness degree of industries increased rapidly and surpassed that of education, indicating that the coordination of industrial development in Chongqing during this period was better, and contributed more to the integration of industry and education. The results are shown in Fig 1.

#### (2) Performance and development trend of the industry-education complex system

Similarly, the coupling degree C and coupling synergy degree D of the industry-education composite system can be calculated as shown in Table 5.

**Table 5:** Coupling coordination level of industry-education composite system.

Year	2000	2001	2002	2003	2004	2005	2006	2007
Coupling degree	0.4987	0.4629	0.4748	0.4834	0.4747	0.4706	0.4659	0.4881
Coupling coordination degree	0.3398	0.3214	0.3191	0.3209	0.3097	0.2982	0.3251	0.3565
Year	2008	2009	2010	2011	2012	2013	2014	2015
Coupling degree	0.4970	0.4971	0.4999	0.4988	0.4887	0.4997	0.5000	0.4999
Coupling coordination degree	0.3841	0.3871	0.4220	0.4426	0.4609	0.4534	0.4750	0.4917
Year	2016	2017	2018	2019	2020	2021	2022	—
Coupling degree	0.4989	0.4986	0.4988	0.4989	0.4997	0.4974	0.4992	—
Coupling coordination degree	0.5040	0.5202	0.5315	0.5564	0.5602	0.5812	0.5722	—

**Fig 2:**
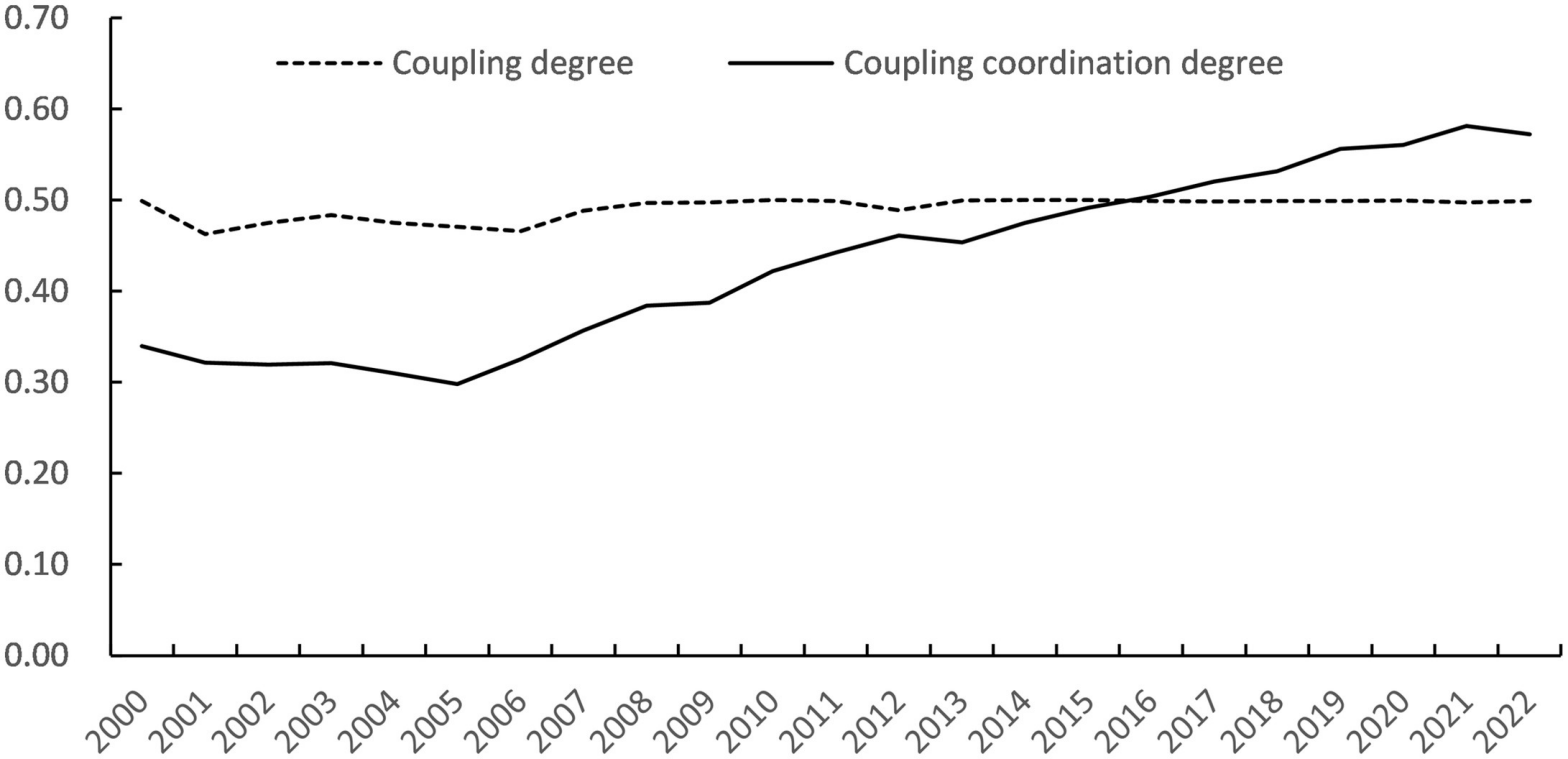
Coupling collaborative development trend of industry-education complex system.

To assess the collaborative development level and trend of industry-education integration of higher education in Chongqing, it is necessary to analyze from two aspects: the coupling coordination degree and coupling degree. Overall, from 2000 to 2022, the collaborative development of higher education industry-education integration in Chongqing has shown a continuous upward trend, but the degree of industry-education coupling has stagnated amid fluctuations, as shown in Fig 2.

In fact, with the joint efforts of the government, enterprises, schools, industries, and all sectors of society, the integration of higher education and industry has gradually deepened in Chongqing, and the level of collaborative education between schools and enterprises has been continuously improved. During the 13th Five Year Plan period, the Municipal Party Committee and Government incorporated the integrated development of higher education industry and education into the overall planning of industrial development, prioritizing education and integrating talents into various policies. In conjunction with the implementation of national major strategies such as innovation driven development, new urbanization, and the strategy of building a strong manufacturing country, they formulated policies for the integrated development of industry and education, committed to building an innovation ecosystem that closely coordinates higher education institutions with industry backbone enterprises and small and medium-sized entrepreneurial enterprises, enhancing the ability to gather talent resources and drive industrial upgrading. The municipal government continued to promote the transformation of ordinary undergraduate institutions into applied technology oriented higher education institutions, and supported the transformation of higher education institutions to carry out professional degree graduate education. Encourage higher vocational schools with conditions to cooperate with applied technology undergraduate colleges to cultivate high-quality technical talents, promote interactive development between higher education and industry, and so on. These measures have effectively promoted the industry-education integration in school-enterprise cooperation. However, due to multiple factors such as institutional mechanisms and policy environments, the coupling intensity of industry-education integration in higher education has stagnated, and the quality of industry-education integration needs to be improved. Some disciplines and majors in applied universities were misaligned with the layout of industrial development, and there was a disconnect between the supply side of talent training and the demand side of industrial development. Enterprises were not actively involved, lacking an effective incentive and restraint mechanism. The form of school-enterprise cooperation was loose, and industry enterprises did not participate deeply enough. The overall faculty in applied universities was weak, with insufficient practical teaching capabilities and outdated infrastructure, which still falls short of national standards. These issues hindered the further deepening of industry-education integration.

## 4. Conclusion and suggestions

### 4.1 Conclusion

Using synergy theory, an industrial-educational composite system and a performance evaluation model for industry-education integration were constructed. Based on the "structure-conduct-performance" analytical paradigm, the indicator system was decomposed. Taking the statistical data of Chongqing from 2000 to 2022 as a sample, the performance of industry-education integration in higher education was evaluated, and its development trend was analyzed. The research results showed that in the long run, the construction effectiveness of the industry-education integration in higher education in Chongqing was obvious, and the collaborative level was constantly rising. The orderliness of the industry subsystem and the education subsystem continued to rise alternately, jointly promoting the continuous increase in the collaborative degree of the industry-education complex system. However, the coupling intensity between industry and education was not high, and the coupling level stagnated, which restricted the further deepening of the industry-education integration. From the perspective of collaborative theory, industry-education integration is a systematic project. Therefore, starting from four levels: industry subsystem, education subsystem, industry-education complex system, and external environment, collaborative efforts were made to promote the continuous improvement of performance in the industry-education integration in higher education in Chongqing, achieving sustainable development of industry-education integration.

### 4.2 Suggestions

#### (1) At the industrial subsystem level, release the effective demand of industrial development for higher education, and improve the enthusiasm of industry and enterprises to participate in the integration of industry and education

First, establish an incentive mechanism for industry and enterprises to participate in the industry-education integration and the cooperation between schools and enterprises, encouraging industry and enterprises to participate in the educational activities of colleges and universities with land, patents, talents, funds, technology and other elements, fulfill their responsibilities according to law and enjoy management and decision-making rights, forming an incentive and restraint mechanism where responsibilities, rights, and interests are equal, so as to improve the enthusiasm of industry and enterprises to participate. Second, support enterprises to deeply participate in the educational reform of vocational schools and colleges, encouraging enterprises to participate in various ways in school major planning, talent training scheme formulation, curriculum textbook development, internship training, teacher training, assessment and evaluation, etc., giving play to the role of enterprise demand in driving talent development. Third, explore a new governance structure for enterprises to participate in the industry-education integration, allowing qualified districts and counties and industry departments in Chongqing to promote the joint-stock reform and mixed ownership reform of applied colleges and vocational schools, establishing an organizational model with clear property rights and clear responsibilities. Attract local well-known enterprises and international and domestic famous enterprise groups to participate in school-enterprise cooperation, jointly establishing secondary industry colleges such as Internet plus, artificial intelligence, information technology around the industrial layout.

#### (2) At the education subsystem level, improve the supply quality of higher education to industrial development, and meet the demand for talents and services in industrial development

First, application-oriented colleges and universities should, based on their own school-running characteristics and resource advantages, explore various forms of industry-education integration collaborative education models to improve the quality of talent cultivation. It is necessary to guide institutions, enterprises, government departments, social organizations and other cooperative entities to jointly invest in funds, technology and other elements, jointly build disciplines, talent cultivation bases and experimental training facilities, and form a joint education model. Second, production and operation practice activities should be integrated into teaching links, and a collaborative education system that integrates theory and practice should be constructed to improve the quality of education. By learning from the successful experience of domestic and international industry-education integration models such as the German dual system, British alternating work-study program, French apprenticeship system, and domestic industry-university-research cooperation, we should continue to explore the school-enterprise alternating integration talent cultivation mode, and clarify the dual identity of student apprentices. The theory-practice-theory alternating should be applied to the entire process of talent cultivation such as course learning, experimental training, skill competition, graduation design. The third is to integrate industry and education to build a " double-qualified" teaching staff, promote two-way talent flow, and improve the quality of the teaching staff. Carry out the evaluation of "double-qualified" titles for university teachers, enterprise engineers, economists, accountants, etc., and improve the salary, benefits, and allowances for dual teacher titles. Encourage university teachers to work part-time in industries and enterprises, and engage in production and operation work such as technology, management, and management in specific positions to gain practical experience. Support enterprise management, technology, and business personnel to work part-time as practical mentors in higher education institutions, carry out practical teaching, and obtain part-time honorary titles and economic rewards.

#### (3) At the industry-education complex system level, promote the interactive coupling of supply and demand between various industries and higher education, and promote the collaborative development of industry-education integration

First, establish a mechanism for interaction between school-enterprise teachers and students, where university teachers serve as enterprise consultants or enter the industry for temporary positions, and enterprise professionals are employed, taught, or trained in universities. Students learn interactively and complementarily in both on-campus classes and off-campus bases, forming an interactive education model. Schools and enterprises, as the supply and demand sides of talent cultivation, directly sign a commissioned cultivation agreement, where enterprises supervise and participate in all aspects of the cultivation process, and sign an employment agreement with students after the completion of the cultivation, forming a commissioned cultivation model that aligns supply and demand. Second, promote the alignment of supply and demand through the industry-education integration, where higher education institutions collaboratively promote the construction of disciplines and specialties in response to industry needs. The discipline and specialty system is adjusted in a timely manner to meet the needs of Chongqing’s modern manufacturing industry, strategic emerging industries, and strategic emerging service industries. Prioritize support for the construction of disciplines and specialties in artificial intelligence, Internet technology, information technology, big data, etc., which are urgently needed in Chongqing at this stage. Vigorously develop specialized disciplines such as smart manufacturing, high-end equipment, new energy, new materials, biomedicine, energy conservation and environmental protection, as well as intelligent logistics and integrated supply chain management. Third, plan collaboratively for industrial development and higher education, optimizing the layout of integrated industry-education development. Integrated industry-education development in higher education is included as an important content in the industrial development planning system, optimizing the spatial layout of integrated industry-education development, guiding outstanding talents to concentrate in industrial and population agglomeration areas, supporting high-quality educational resources to serve the rural revitalization strategy, and integrating with the primary industry in rural areas.

#### (4) At the external environment level, optimize the support and guarantee environment for the integration of higher education and industry, and create a good atmosphere for the integration of industry and education through multi-party collaboration

First, the linkage of fiscal, financial, and insurance policies to enhance the level of protection, increase financial support for applied colleges and universities, and create a policy environment for the industry-education integration. Increase financial investment in the industry-education integration, improve the infrastructure conditions and talent cultivation environment for the industry-education integration in applied colleges and universities. For the industry-education integration projects, support financial departments such as banks to develop diversified financing products to provide financial support for the industry-education integration. Establish liability insurance and personal accident insurance for productive practices such as off-campus student internships and off-campus base training, and encourage commercial insurance companies to develop specialized supporting systems for modern apprenticeships and new apprenticeship systems in enterprises. Second, the linkage of government, enterprises, and schools to implement demonstration projects of the industry-education integration, creating a good atmosphere for the industry-education integration. Taking the national industry-education integration project as an opportunity, promote the transformation construction pilot and demonstration project of high-level applied undergraduate colleges and universities in our city, and exchange successful experiences in the industry-education integration. For different levels of development in different districts and counties, carry out pilot projects for the construction of demonstration districts and counties for the industry-education integration in a hierarchical and step-by-step manner. Establish a performance evaluation system for the industry-education integration, formulate incentives for the industry-education integration, and play a role in benchmarking guidance and demonstration effects.

## Supporting information

S1 Dataset (XLSX)
